# Essential Clinical Global Health, edited by Brett D. Nelson

**Published:** 2017-04-20

**Authors:** Shefali Thakore, William P. McKay

**Affiliations:** 1 Department of Anesthesiology, University of Saskatchewan, Saskatchewan, Canada

## Abstract

In the past two decades there has been a more concerted effort to make rational, evidence-based approaches to what has been, for the previous two centuries, a somewhat chaotic mixture of missionary-based, NGO-based, university and United Nations-based efforts at international research and clinical practice in financially poor countries. *Essential Clinical Global Health* is a welcome new addition to problems of providing good quality health care in resource-limited settings. The emphasis is on poor countries, although some information is useful for remote settings in developed countries.

*Essential Clinical Global Health*, edited by Brett D. Nelson^1^ is yet another addition to the popular and successful Essential series of textbooks. These textbooks are noted for their coverage of the basics of a variety of topics in health sciences.

*Essential Clinical Global Health* brings us the emerging topic of clinical work in resource-limited settings so ubiquitous in poor countries referred to by the World Bank by the awkward name of Low- or Middle-Income Country (LMIC). The organization of the textbook is well laid out in six parts with each part dedicating chapters to pertinent topics. Key learning objectives listed at the beginning of each chapter are complemented by numerous flow charts, clinical pearls, clinical experiences, case studies, and red flags scattered throughout the textbook. These highlighted areas along with figures, tables, and boxes make this a palatable read. The reference list is solid with data and guidelines supported by experts in international health organizations such as the World Health Organization (WHO), United Nations Children’s Emergency Fund (UNICEF), US Centers for Disease Control and Prevention, American Colleges (Obstetrics and Gynecology, Trauma, etc), and various authors who have published articles in high impact journals such as the New England Journal of Medicine. The editorial board is American with one Australian, and the textbook appears to be aimed primarily at healthcare workers from wealthy countries who volunteer to work in resource-limited settings. The authors, mostly from the Boston area, are experts principally in pediatrics, infectious diseases, and emergency medicine. Of note, there are very few collaborative efforts made with experts from other countries: of 97 authors, only nine are from LMICs. Most core textbooks of clinical care in developing nations, including this one, cover newborn care, pediatrics, maternal health, and infectious diseases well; however, topics in surgery, critical care, pain management, and mental health in resource-limited settings are scant or absent.

From our background in anesthesia/trauma and experience volunteering in Rwanda, we found the chapters on trauma and emergency care (chapters 25 and 26, respectively) covered the basics of the Advanced Trauma and Life Support (ATLS) course but not much more. A progressing area of trauma management is the use of Focused Assessment with Sonography (FAST), which is mentioned but not described. The use of ultrasound in trauma is rising in developing nations, and should be more thoroughly described in the next edition. Also disappointing were the chapters’ failure to detail the rising epidemic of injury-related morbidity and mortality. In one of the World Health Organization’s Bulletins (2009), injuries were noted to be a “neglected epidemic in developing countries, causing more than five million deaths each year, roughly equal to the number of deaths from HIV/AIDS, malaria and tuberculosis combined.”^2^ The forecast of trauma related injuries and death in developing nations is dismal as more cars, motorbikes, and other wheeled vehicles congest roads and collide with pedestrian traffic. Emergency care of the trauma patient is complex involving prompt resuscitation with fluids and blood, and often, immediate transfer to the operating room. There are many limitations of transfusion medicine in resource limited settings and a brief discussion of this would be appropriate in a chapter on trauma. Blood is often available but other essential blood products can be difficult to procure. The operating room is a unique setting and a short analysis of some of the challenges would be beneficial. Another oversight is how developing nations deal with pain. Pain management and its complement of rehabilitation medicine remains inadequate for a variety of reasons, none of which are mentioned in this primer on global health. Analgesic options are few but many countries are developing strategies to effectively deal with pain.

This textbook wisely advocates the need to be respectful and work collaboratively with community health workers and other allied health professionals such as nurses. However, there have been considerable strides made in integration of local medical students, residents and attending physicians with visiting physicians from more developed nations. These partnerships have been critical in improving established curricula and developing new ones. As well, basic medical simulation labs are being set up to facilitate hands-on learning for both nurses and medical students/residents. There are many examples of this and highlighting these partnerships would help readers understand that the ultimate goal for developing nations is one of self-sufficiency and sustainability in training of health care deliverers at a higher level.

In terms of developing a career in global health, this textbook covers the growing popularity of post residency fellowship training or courses in global health. However, it should be emphasized that the majority of physicians do not have global health fellowships and are involved in one of three types of volunteering - missionary work (e.g., Mercy Ships), educator/mentorship/research (e.g., Health Volunteers Overseas), or crisis/emergency relief (e.g., Doctors Without Borders). This mosaic of volunteer opportunities in global health has evolved to include both new and experienced physicians, especially those in sub-specialities and surgical fields, to join their cohorts in primary care.

The Wiley E-Text version of the textbook is easy to use and as an electronic reference is portable on a variety of devices. The companion website containing additional resources such as videos and multiple choice questions is beneficial in consolidating knowledge found in the textbook.

As a primer of global health, *Essential Clinical Global Health* covers the requisites well except for some new areas of collaborative and progressive higher-level education programs. From a recent editorial in the journal *Anesthesiology*:

Many in global health discounted the surgical agenda for decades, claiming that surgery was too expensive and “a luxury” in countries suffering from infectious disease. Experts in global health also suggested that surgical disease, which includes every organ system and crosses all age groups and equally impacts both sexes, was “too complicated” to address in resource-constrained environments. All of these historical arguments have been effectively refuted—surgery and safe anesthesia are cost-effective and will prevent disability and premature death, saving families and governments’ billions of dollars if applied responsibly. Regardless of the complexities involved, if human immunodeficiency virus/acquired immunodeficiency syndrome can be addressed in low-and-middle-income countries so can surgical care and safe anesthesia.

We look forward to the inclusion of these issues in future editions.
